# Effect of minimally invasive posterior veneer Preparation designs on failure load and mode after fatigue (in-vitro study)

**DOI:** 10.1186/s12903-025-06457-6

**Published:** 2025-07-26

**Authors:** Islam Mohamed Mady, Ahmed Safwat El Kady, Moustafa Nabil Aboushelib, Mohamed Hussein A. Hussein

**Affiliations:** 1 Conservative Dentistry Department, Department of Conservative Dentistry Department, Faculty of Dentistry, Alexandria University, Champolion St., Azarita, Alexandria, 21527 Egypt; 2 Biomaterials Department Faculty, Faculty of Dentistry, Alexandria University, Alexandria, 21527 Egypt; 3 Conservative Dentistry Department, Department of Conservative Dentistry Department, Faculty of Dentistry, Alexandria University, Alexandria, 21527 Egypt

**Keywords:** Ceramic veneer, Minimally invasive treatment, Premolar, Fatigue test, Aesthetics

## Abstract

**Background:**

Ceramic veneers are a common option for enhancing dental aesthetics by improving tooth form, shade, and function. Preparation design is considered a critical factor in the success of dental veneers, as it influences the failure load and fatigue survival of posterior minimally invasive ceramic veneer restorations. The purpose of the study is to compare the failure load and mode after fatigue among three types of veneer preparation designs for restoring premolars.

**Methods:**

64 human premolars were equally divided into four groups. Group 1 had a buccal preparation depth of 0.7 mm; Group 2 had an additional reduction of the buccal cusp; Group 3 had a mesio-occluso-distal box with dimensions of 2 mm wide and 3 mm deep. The remaining sound teeth served as the control group. Monolithic lithium disilicate restorations were cemented onto the prepared teeth with adhesive cement and then exposed to dynamic fatigue and thermocycling. Each specimen underwent a single load-to-failure test, and the discrepancy in failure load before and after fatigue was computed.

**Results:**

No significant differences in initial failure load were observed among the four groups evaluated (*p <*.075). A significant drop in failure load was observed in all groups after fatigue (P = 0.012). The failure loads of Groups I and II were significantly lower than those of Group III and the control (P < 0.05).” Nevertheless, after fatigue, statistically significant differences were detected (*p <*.013) as fatigue caused a substantial drop in failure load among all groups.

**Conclusions:**

Within the limitations of this study, fatigue significantly reduced failure load in all groups. Veneer preparation design influenced post-fatigue strength, with more conservative designs showing better resistance. These findings underscore the impact of fatigue on structural integrity and the importance of tooth preparation design on fatigue resilience.

## Background

Aesthetic dentistry and cosmetic rehabilitation are considered one of the most challenging procedures in clinical dental practice. An aesthetic smile can often be achieved using dental veneers, inlays, and onlays, which restore both aesthetics and function [1]. Recently, the “minimally invasive concept” has been advocated to preserve tooth structure whenever possible. Less invasive options are now available due to the newly introduced adhesive systems and high-strength ceramics, which provide the same effectiveness as complete coverage restoration. Dental ceramic laminate veneers are a reliable, functional, and aesthetic treatment option in the anterior dentition [2]. Occlusal veneers and onlays aim to replace the occlusal tooth material absent in the posterior region [3–5]. Since 92–97% of people can see maxillary premolars when they smile, restorations for these teeth must meet the strictest aesthetic standards [6, 7].

VOnlay is a unique concept that combines an Onlay with an extended buccal veneer. Instead of using full coverage restorations, VOnlay is specifically designed for use in bicuspid regions [8]. Dental literature has shown a strong link between strength deterioration and the loss of tooth structure [9]. Full-veneer restorations are now being utilized as a less invasive alternative to full-coverage crowns. They effectively cover the occlusal, labial, and proximal areas, addressing any defects that may be present [10, 11]. Lithium disilicate (LDS) glass-ceramic, produced using press or CAD/CAM technologies, exhibits favourable aesthetic and mechanical properties appropriate for single-tooth restorations [12, 13]. Furthermore, there have been publications suggesting positive clinical outcomes and high rates of long-term survival and success for various dental procedures, such as posterior inlays, anterior veneers, onlays, overlays, and crown restorations [14, 15].

Due to the favourable clinical results, the manufacturer has revised the recommendation for adhesively cemented Lithium Disilicate Glass (LDS) crown restorations to a thickness of 1 mm [16]. Recently, occlusal veneers or table tops have been developed for the molar region. These veneers are extremely thin, comparable to Onlays, and have no retentive features. They provide a conservative alternative to traditional full-coverage crowns [17, 18]. Nevertheless, there is currently a lack of clinical and in vitro evidence regarding posterior defect-oriented preparation forms, such as full-veneer restorations with reduced restoration thickness [19].

The preparation geometry for all-ceramic restorations often adheres to the conventional principles employed for metal-cast gold preparations. These criteria encompass integrating retentive features, such as an occlusal box preparation [20]. The recommended ceramic thicknesses for the occlusal surface range from 1.0 to 2.0 mm, while for the buccal aspect, they range from 0.3 to 1.0 mm. The specific all-ceramic system determines the exact thickness within these ranges [16]. For the premolars, the guidelines for the preparation of defect-oriented posterior full-veneer restorations remain scarce. Based on the current literature, the risk of failure for all-ceramic veneers significantly increases when the preparations expose dentin or show a deficiency of enamel in the cervical region [21, 22].

This in vitro study aimed to compare the failure load and mode after fatigue among 3 types of veneer preparation designs for restoring premolars. The null hypothesis proposed no significant differences in failure load among the suggested preparation designs.

## Methods

### **Sample** size calculation

The primary outcome of the present study is failure load.

The mean ± SD failure load **(primary outcome)** was 1005 ± 338 N for full veneer preparation with box [23], 983.56 ± 202 N for the full veneer preparation with occlusal preparation [24], 415.9 ± 147.2 N for the labial veneer preparation [25], and 949.6 ± 331.5 N for the sound unprepared teeth [26]. Sample size is estimated assuming level of significance 5% (α error accepted = 0.05) and a power of 80% (β = 0.20). Based on the difference between independent means using the highest SD = 383 to ensure enough power. The minimum sample size was calculated to be **8 samples** per group. Total sample = number per group x number of groups x number of subgroups = 8 × 4 × 2 = **64 samples** [27]. Any sample loss from the study sample due to processing error will be replaced to maintain the sample size [28]. The sample size was calculated using GPower version 3.1.9.2 [29].

### Sample Preparation

Sixty-four recently extracted intact maxillary premolars were obtained from patients who sought treatment at the Oral Surgery Department of the Faculty of Dentistry, Alexandria University. The teeth were thoroughly cleansed and preserved in distilled water. The teeth were first cleaned using an ultrasonic scaler. They were then immersed in a Chloramines-B-hydrate solution for seven days, eliminating all types of microorganisms without affecting the hardness or microstructure of the enamel or dentin. Afterward, the teeth were preserved for 90 days in distilled water [30]. The roots of all teeth were immersed in molten utility wax to form a 0.2–0.3 mm thick layer to simulate the periodontal ligament space. Each specimen was embedded in a self-curing acrylic resin 2 mm below the cementoenamel junction using a split metallic copper mold of 20 mm in length and 14 mm in diameter [31]. The wax spacer was removed from the root surface, and then polyether adhesive was coated on the roots until thoroughly dried. Polyether impression material coated the roots entirely for the simulation of the periodontal ligament, and then they were returned to their acrylic resin space (alveolus) and allowed to be set. The surplus polyether material was excised using a scalpel blade to create a level surface for each tooth [32]. The blocks were removed from the split mold after the acrylic resin was set on each tooth.

A dental imprint of all the teeth to standardize the preparation was taken using vinyl polysiloxane material (3 M ESPE Vinyl Polysiloxane (VPS) Seefeld Germany). Before starting the procedure, accurate silicon indices of each tooth were collected to evaluate the degree of reduction. The indices were engraved in both bucco-palatal and meso-distal orientations to function as a silicon index or guide for restoring the tooth to its natural anatomical form. The indices protruded a minimum of 3 mm beyond the cervical region of the tooth’s crown to guarantee precise placement of the mold during each substitution [18, 33].

**Fig. 1:**
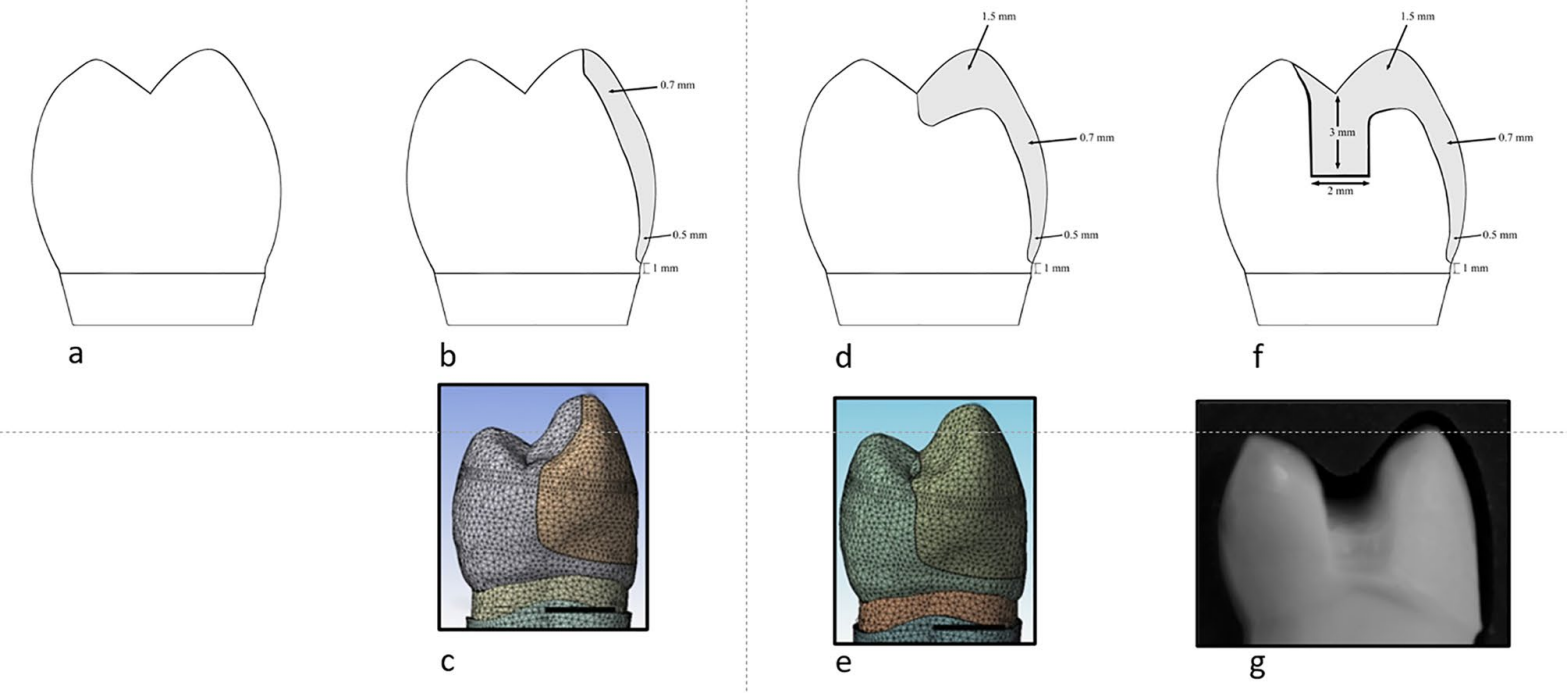
Schematic presentation of the different veneer preparation designs. (**a**) Schematic diagram of sound unprepared premolar (**b**) Schematic diagram of buccal surface veneer preparation (**c**) 3-D model of buccal surface veneer preparation (**d**) Buccal surface veneer preparation with buccal cusp preparation (**e**) 3-D model of buccal surface veneer preparation with buccal cusp preparation (**f**) Buccal surface veneer preparation with buccal cusp and MOD box preparation (**g**) 3-D model of buccal surface veneer preparation with buccal cusp and MOD box preparation

For all groups, designs were created using a diamond stone for veneer preparation (Microdont, Brazil). The specimens were randomly sorted into four primary groups, each consisting of 16 specimens. Schematic presentation of the different veneer preparation designs used in the present study is illustrated in Fig. 1.

**Fig. 2:**
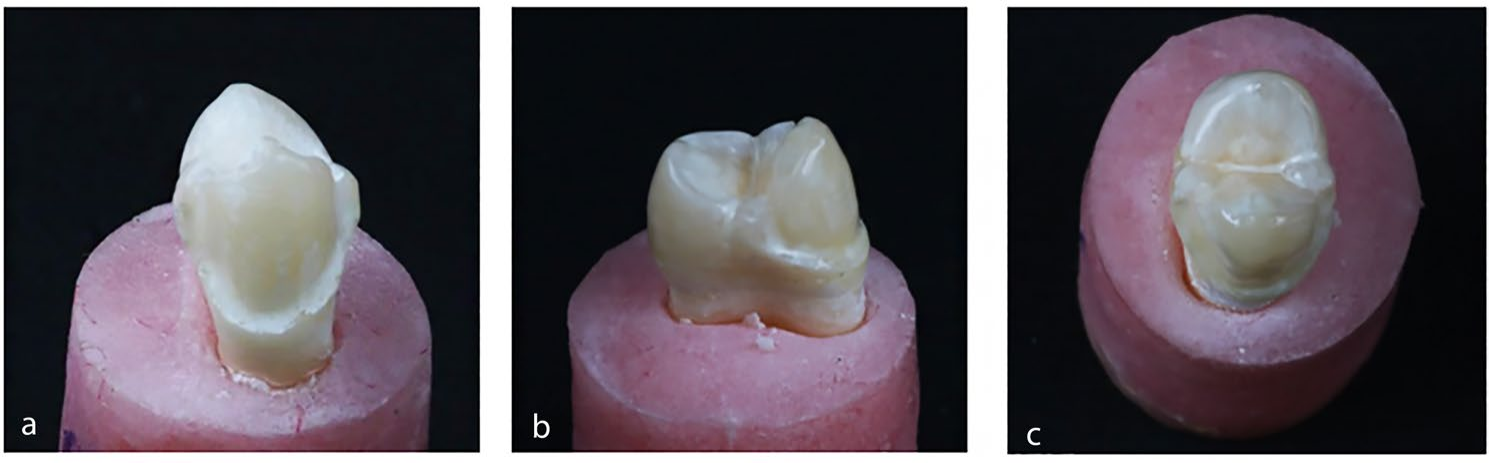
Buccal surface preparation. (**a**) Buccal view, (**b**) Proximal view, (**c**) Occlusal view

**Fig. 3:**
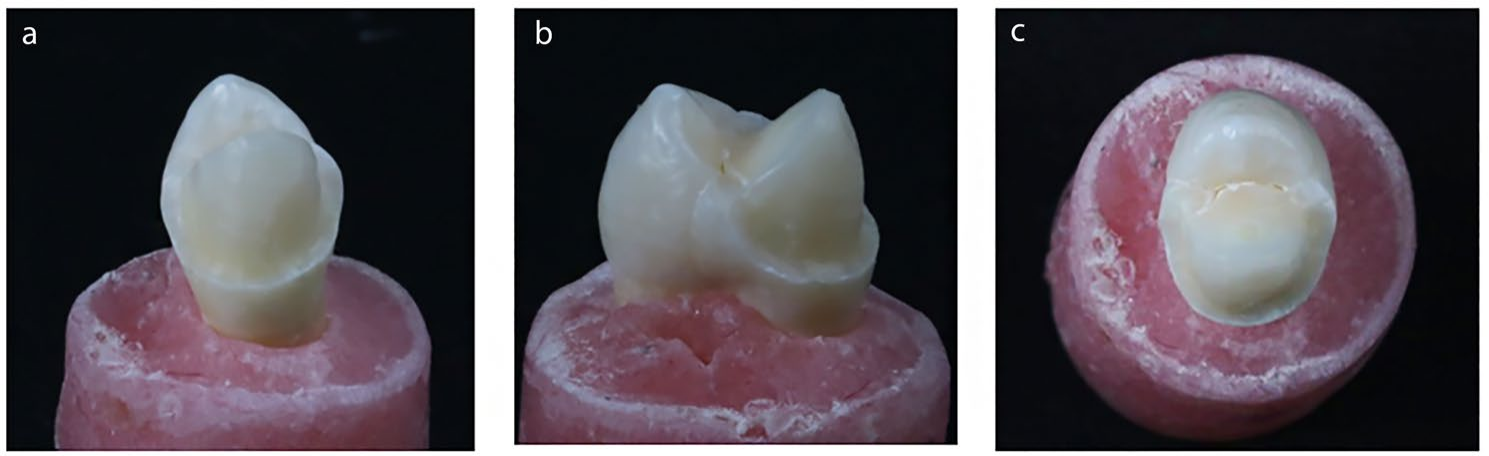
Buccal surface with buccal cusps preparation. (**a**) Buccal view, (**b**) Proximal view, (**c**) Occlusal view

**Fig. 4:**
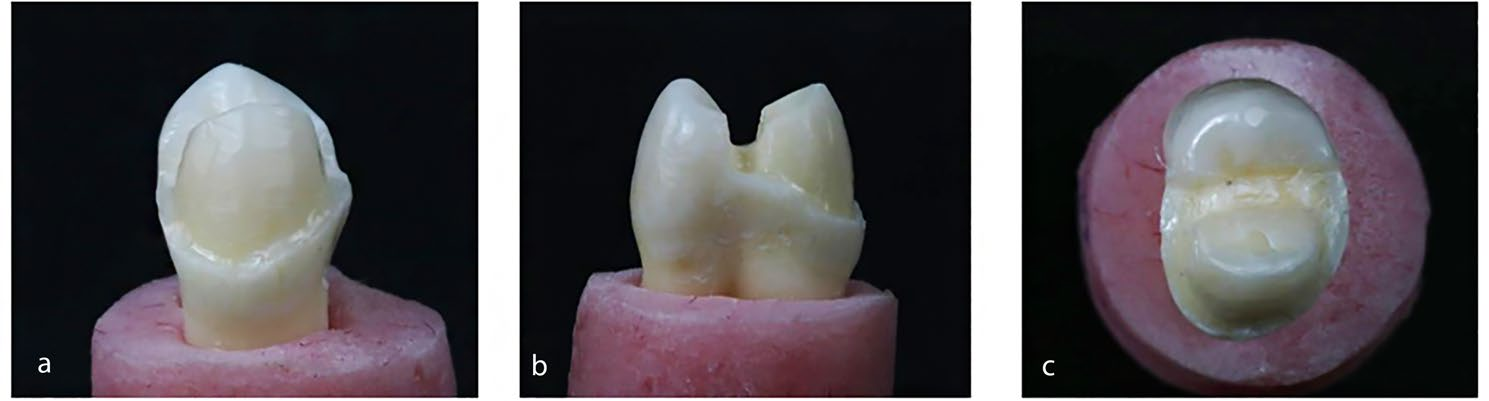
buccal surface with buccal cusp preparation and MOD box preparation. (**a**) Buccal view, (**b**) Proximal view, (**c**) Occlusal view

The control group was sound unprepared premolars. Group I (buccal surface veneer preparation), a chamfer finish line with a width of 0.5 mm was prepared 1 mm above the cementoenamel junction on the buccal surface of the tooth. The buccal reduction was set at a depth of 0.7 mm (Fig. 2). Group II (buccal surface veneer preparation with buccal cusp preparation) was prepared with a 0.7 mm buccal reduction and buccal cusp reduction with a depth of 1.5 mm extending palatally to the central groove (Fig. 3). Group III (buccal surface veneer preparation with buccal cusp and MOD box preparation) was prepared with a 0.7 mm depth of buccal reduction, buccal cusp reduction with a depth of 1.5 mm, and a Mesio-Occluso-Distal (MOD) box preparation. The occlusal box is prepared with 3 mm depth and 2 mm width. The proximal box had a depth of 1.5 mm and 3 mm width (Fig. 4).

A dental laboratory scanner (Medit i710) digitized the prepared teeth to capture digital impressions, with the teeth sprayed (Cerec optispray) to improve the precision of the digital impression.

A Computer-Aided Design (CAD) software (Exocad, Exocad GmbH in Darmstadt, Germany) was utilized to design the lithium disilicate restorations (IPS e.max CAD, Ivoclar Vivadent in Schaan, Liechtenstein). The design was sent to CAM milling equipment (CEREC 3, Sirona Dental Systems, GmbH in Bensheim, Germany). Computer-Aided Design (CAD) and Computer-Aided Manufacturing (CAM) (CAD-CAM) blocks, A3 in the shade and C14 in size, were inserted into the machine and subjected to wet milling followed by crystallization heat treatment (Programmat P310, Ivoclar Vivadent, Schaan, Liechtenstein).

### Adhesive cementation

A dual cure cement (Bisco DUO-LINK, Bisco USA) was selected for the cementation of the restorations. Before applying adhesive cement, the inner surfaces of the restorations were exposed to 9.5% hydrofluoric acid (Ceramic Etchant; Bisco, USA) for 30 s. Subsequently, they were rinsed entirely using an air-water spray for 30 s, followed by air drying. The fitting surface was coated with a precise application of a single layer of silane coupling agent (Bisco, USA) using fine brushes for 60 s, followed by air drying.

The tooth surface was etched with a 37% phosphoric acid solution for 20 s, washed, and then softly dried using air. A layer of adhesive was applied and subjected to light curing for 20 s. A thin layer of dual-cure resin cement was applied, and the restorations were seated gently with finger pressure. Any surplus cement was eliminated, and the Veneer was briefly exposed to a low-intensity light source for 2 s to facilitate removing any remaining excess cement. The cement was lightly polymerized for 40 s.

### Initial and residual fracture strength test

**Fig. 5:**
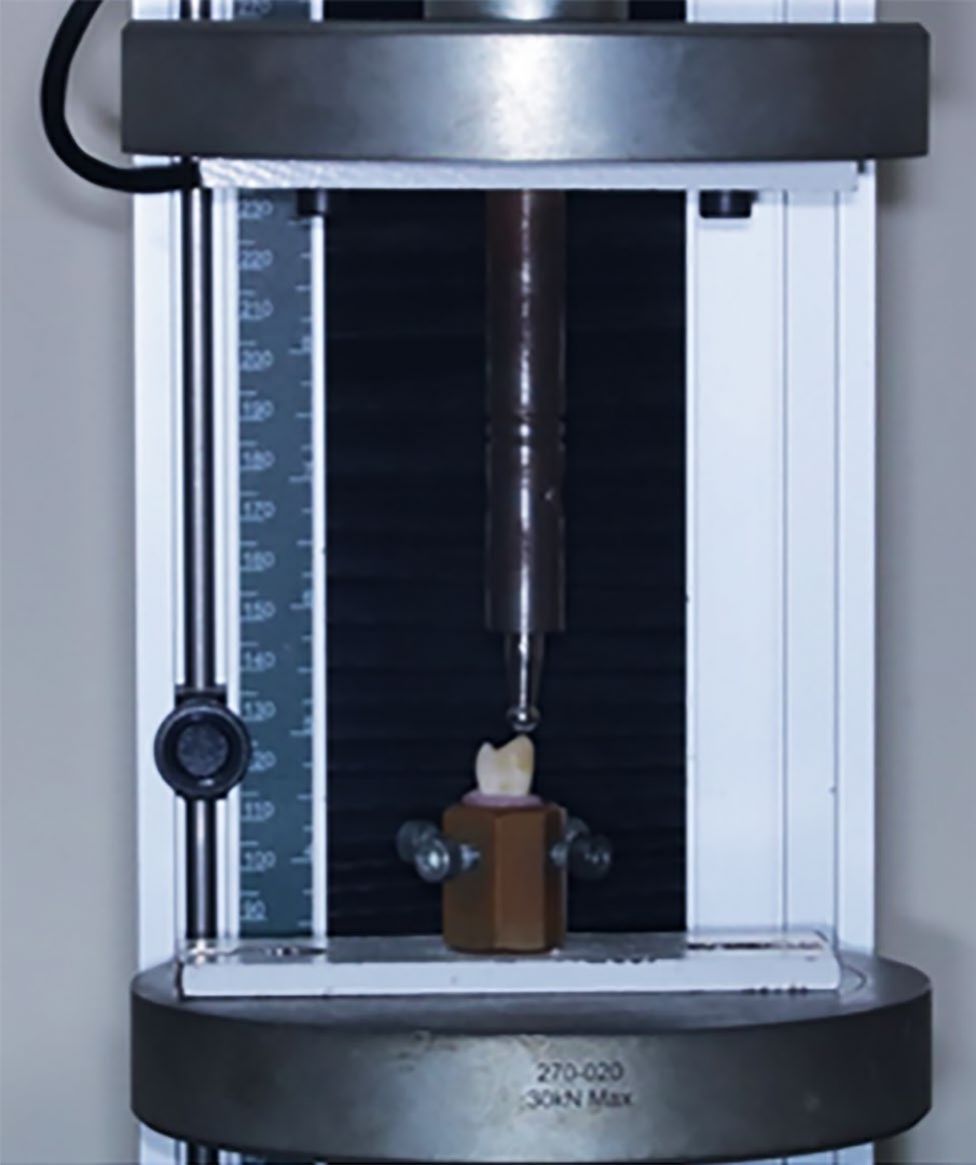
Universal testing machine (5 ST, Tinius Olsen England 2018)

Half the restorations received one cycle load to failure using axial loading in a universal testing machine (Tinius Olsen, 5 ST, England) (Fig. 5). The experiment was performed with a steel ball of five mm in diameter, with the crosshead speed set at 1.5 mm/min [34]. The indenter was positioned at the central fossa with a plastic stress breaker. The specimens were subjected to axial stress until they fractured, and the highest load at which failure occurred was measured and analysed using appropriate software.

The other half of the specimens underwent cyclic loading (1.2 mil cycles, with a force range of 100–150 N, at 5 s contact time) using an ACTA-type pneumatic fatigue tester [35]. To replicate the natural chewing process, the palatal cusp was subjected to cyclic fatigue directed toward the central fissure. The test was performed in a water bath under a 5–55 degrees Celsius temperature cycle. Cracks, fractures, or debonding were examined in the specimens after fatigue testing.

#### Failure analysis

The specimens were visually inspected using a light stereomicroscope (model SZ1145TR, Olympus Japan 1990) at magnifications of 5 and 10 times, and failure modes were categorized as follows: **Type I**: minimal repairable crack propagation in the ceramic material; **Type II**: Cohesive unrepairable fracture in the ceramic material, leaving the tooth intact; **Type III**: Catastrophic fracture of restoration involving part of the tooth structures, **Type IV** Catastrophic fracture of the tooth and root [9].

### Statistical analysis

Data were collected and entered into the computer system using Statistical Package for Social Science (SPSS) software (version 25) [36]. Shapiro–Wilk normality test to assess the distribution of the quantitative variables proved significant [37], so non-parametric statistics were utilized [38]. The Kruskal-Wallis test evaluated differences in failure load and percent change [39], followed by Dunn’s post hoc test [40, 41]. The *p*-value was adjusted using the Bonferroni correction method [42]. The tests were two-tailed, with a significance level set at a *p*-value of < 0.05.

## Results

There were no statistically significant differences in initial failure load (*p* =.075) among the tested groups. However, after fatigue, significant differences were detected (*p* =.013). Significant reduction in failure load was observed after fatigue among the four studied groups (Control: *p =*.012), (Group I: *p =*.012), (Group II: *p =*.012), (group III: *p =*.012). The percentage change (%) of failure load after fatigue was statistically significantly different among the studied groups (*p =*.026). Group III showed the least change compared with Group I (*p* =.028) and Group II (*p* =.039). All other pairwise inter-group comparisons were not statistically significant (*p* >.05) (Table 1).

**Table 1:** Comparison of the failure load (N) before and after fatigue in the four studied groups

Failure load (*N*)	Group				Test of significance *p*-value
	Control (*n* = 8) (a)	Group I (*n* = 8) (b)	Group II (*n* = 8) (c)	Group III (*n* = 8) (d)	
**Before Fatigue**					
- Min.– Max. - Median - 95% CI for median - 25th − 75th Percentile	987.24–1410.69 1211.57 1156.23–1309.00 1160.24–1277.16	949.22–1420.82 1061.61 974.56–1318.60 975.68–1263.42	998.78–1227.32 1140.51 1059.95–1203.20 1083.15–1180.76	1011.65–1402.30 1313.85 1136.94–1364.59 1194.80–1353.55	H_(KW)_ = 6.895 *p* =.075 NS
**After Fatigue**					
- Min.– Max. - Median - 95% CI for median - 25th − 75th Percentile	870.23–1236.47 1130.39^**a, b,c, d**^ 997.89–1227.51 1003.56–1213.15	798.36–1265.20 953.20^**a, b,c**^ 872.36–1106.21 882.34–1067.21	874.23–1132.60 966.71^**a, b,c**^ 922.33–1102.30 937.47–1047.33	979.15–1389.25 1243.22^**a, d**^ 1025.11–1298.25 1096.67–1296.86	H_(KW)_ = 10.739 *p* =.013^*^
**Test of significance** ***p-value***	Z_(WSR)_ = 2.521 *p* =.012^*^	Z_(WSR)_ = 2.521 *p* =.012^*^	Z_(WSR)_ = 2.521 *p* =.012^*^	Z_(WSR)_ = 2.521 *p* =.012^*^	
**Percentage Change (%)**					
- Min.– Max. - Median - 95% CI for median - 25th − 75th Percentile	-14.46– -1.14 -12.10^**a, b,c, d**^ -14.29– -1.24 -13.50– -3.73	-22.02– -2.84 -10.82^**a, b,d**^ -18.92– -5.45 -17.71– -6.95	-20.21– -7.72 -12.85^**a, c,d**^ -14.33– -8.39 -13.90– -9.26	-10.10– − 0.93 -4.17^**a, b,c, d**^ -9.84– -2.47 -7.35– -2.84	H_(KW)_ = 9.241 *p* =.026^*^

n: Number of patients

Min-Max: Minimum– Maximum

CI: Confidence interval

H_(KW)_: Kruskal-Wallis Test

Post-hoc comparison:

*Superscript letters (a) thru (d) are assigned to groups in order*

*Different superscript above the median indicates statistical significance (for example if control group median carries the letter (a) (by default) and (d)… this means no statistical significance between control and group III).*

*i.e. for After fatigue Only*,* group III had statistical significance against Group I and Group II.*

*For percentage change only group II had statistical significance against group III.*

*: Statistically significant (*p* <.05)

NS: Statistically not significant (*p* ≥.05)

Comparisons were carried out among groups using Kruskal-Wallis test.(Kruskal & Wallis, 1952) Post-hoc pair-wise comparisons when Kruskal-Wallis test was significant was carried out using Dunn-Sidak test for multiple comparison.(L. K. Dunn et al., 2020; O. J. Dunn, 1964) *p* value was adjusted using Bonferroni correction method.(Schober & Vetter, 2020)

Dunn, L. K., Taylor, D. G., Chen, C.-J., Singla, P., Fernández, L., Wiedle, C. H.,.. Shaffrey, C. I. (2020). Ventilator mode does not influence blood loss or transfusion requirements during major spine surgery: a retrospective study. *Anesthesia & Analgesia*,* 130*(1), 100–110

Dunn, O. J. (1964). Multiple comparisons using rank sums. *Technometrics*,* 6*(3), 241–252

Kruskal, W. H., & Wallis, W. A. (1952). Use of ranks in one-criterion variance analysis. *Journal of the american statistical association*,* 47*(260), 583–621

Schober, P., & Vetter, T. R. (2020). Adjustments for multiple testing in medical research. *Anesthesia & Analgesia*,* 130*(1), 99

**Fig. 6:**
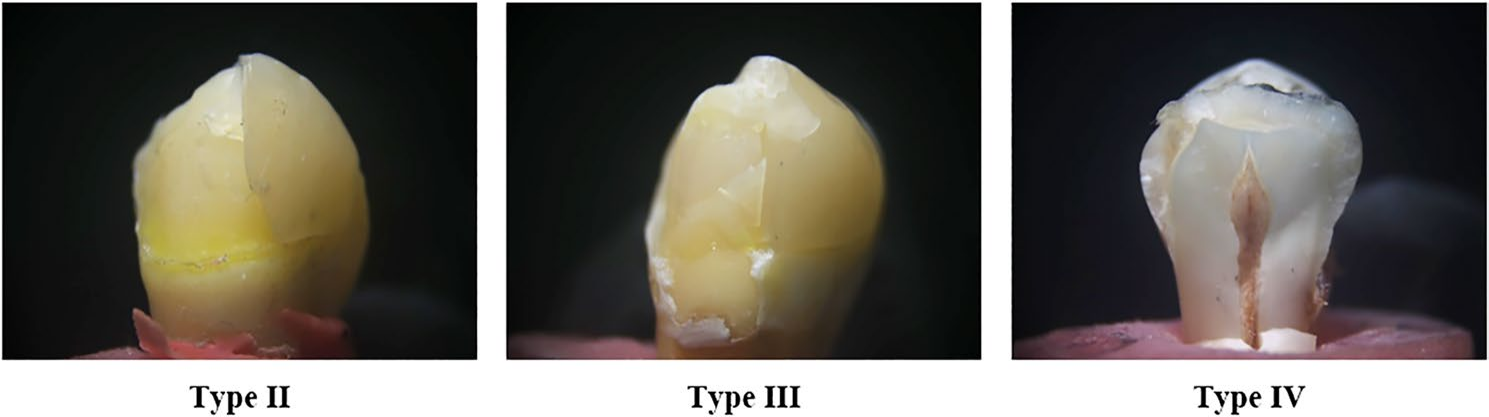
Different modes of failure occurred in the specimens of the present study (magnification x18)

There were no significant differences in the failure mode between the tested groups (*p* <.069). The predominant failure mode was class II, characterized by cohesive fracture inside the ceramic material while keeping the tooth intact. This kind of failure occurred in 50% of cases in group I, 37.5% in group II, and 81.25% in group III. Type IV failure, characterized by root fracture, was detected in 18.75% of group I, 12.5% of group II, and 12.5% of group III **(**Fig. 6**)**.

## Discussion

Based on the statistically significant differences observed (*p* <.05), the null hypothesis was rejected, confirming that preparation design impacts the failure resistance of ceramic veneers after fatigue.

To the author’s knowledge, this is the first study that systematically investigated the non-retentive full-veneer preparation design at different thicknesses on premolars after fatigue.

There is a lack of comprehensive preclinical and clinical evidence regarding premolar restorations. Vonlay restorations are highlighted for their superior aesthetic outcomes and preservation of tooth structure, making them an advantageous choice for posterior teeth afflicted with extensive caries-induced cavities.

We found a significant difference in fracture load but no significant difference in the failure type among the tested designs after fatigue. Schwendimann and Özcan [43] discovered that root fractures, among various types of failures, had minimal or no clinical significance. This phenomenon could be ascribed to pre-existing fissures in the extracted teeth or may have been affected by repetitive stress in the chewing simulator. These findings should be considered alongside the results of this study’s mode of failure analysis. The restored teeth were also subjected to 1,200,000 loading cycles. It was found that applying cyclic loading combined with thermal gradient enhances the simulation of clinical conditions [43]. Future research should include advanced fatigue components in the experimental design to obtain more clinically significant information about the material’s ultimate strength after fatigue, which is also relevant to this study. Nevertheless, the present study did not use acidic stimuli encountered in the oral cavity, human saliva, or synthetic saliva as a medium.

Guess et al. [9] discovered that decreasing the depth of preparation to 1.00 mm and 0.5 mm did not negatively affect the ability of pressable lithium-disilicate ceramic Onlay restorations to withstand fractures. It led to decreased failure loads in the case of full veneer restorations on premolars. Furthermore, it was found that palatal-Onlay restorations exhibited considerably greater resistance to fractures when they had ultra-thin thicknesses compared to conventional thicknesses (*p* =.015). Thickness changes did not have an impact on Onlay restorations. The fracture stresses of conventional full veneers were significantly greater than those of thin and ultra-thin restorations. Guess et al. [9] reported a median load to fracture of 1300 (1130–1532) N, consistent with the present study findings (median: 1243 (1096.67–1296.86). Chang, Yu, & Lin [44] reported a mean fracture load of palatal cusp-replacing ceramic restorations with buccal cuspal coverage of 1559 ± 337 N, which is in agreement with our findings in group III before fatigue.

The limitation of the present study is that the findings are relevant to the specific all-ceramic materials and luting systems examined. Additionally, the use of human teeth introduces some variability due to differences in storage time, hard tissue thickness, and morphological variations among the teeth studied, which may affect the generalizability of the results.

## Conclusions

Within the limitations of this study, it demonstrated that all ceramic veneer preparation designs experienced a significant drop in failure load after fatigue. Notably, the preparation design played a critical role in post-fatigue strength, with Groups III and the control exhibiting superior resistance. These findings suggest that more conservative veneer preparation designs may enhance the long-term durability of restorations. Further in vivo studies are warranted to validate these results and guide clinical protocols.

### Recommendations

Hence, minimally invasive non-retentive LDS full-veneer restorations demonstrated considerable failure loads, particularly those with reduced thicknesses and adhesively cemented and subjected to cyclic mechanical loading with simultaneous thermocycling; it could serve as a viable treatment for addressing buccal mesio-occluso distal MOD defects, particularly in maxillary premolars.

Clinical application of the present findings may be important for guidelines of veneer restoration. It is essential to validate the findings of this in vitro study through further clinical investigations.

## Data Availability

Data is available upon request.

## Abbreviations

LDS: Lithium Disilicate Glass
MOD: Mesio-Occluso-Distal
CAD: Computer-Aided Design
CAD-CAM: computer-aided design (CAD) and computer-aided manufacturing (CAM)
MOD: Mesial Occlusal Distal
USPHS: United States Public Health Service
N: Newton
